# A Mediterranean Diet-Oriented Intervention Rescues Impaired Blood Cell Bioenergetics in Patients with Metabolic Dysfunction-Associated Steatotic Liver Disease

**DOI:** 10.3390/diagnostics14182041

**Published:** 2024-09-14

**Authors:** Agnese Segala, Marika Vezzoli, Alice Vetturi, Emirena Garrafa, Barbara Zanini, Emanuela Bottani, Monica Marullo, Silvia Marconi, Chiara Ricci, Alessandra Valerio

**Affiliations:** 1 Department of Molecular and Translational Medicine, University of Brescia, 25123 Brescia, Italy; agnese.segala@unibs.it (A.S.); marika.vezzoli@unibs.it (M.V.); alice.vetturi@unibs.it (A.V.); emirena.garrafa@unibs.it (E.G.); emanuela.bottani@univr.it (E.B.); 2 Department of Laboratory Diagnostics, ASST Spedali Civili, 25123 Brescia, Italy; 3 Department of Clinical and Experimental Sciences, University of Brescia, 25123 Brescia, Italy; barbara.zanini@unibs.it (B.Z.); monica.marullo@unibs.it (M.M.); silvia.marconi@unibs.it (S.M.); chiara.ricci@unibs.it (C.R.); 4 Division of Gastroenterology, ASST Spedali Civili, 25123 Brescia, Italy

**Keywords:** metabolic dysfunction-associated steatotic liver disease, Mediterranean diet, mitochondrial bioenergetics, peripheral blood mononuclear cells, descriptive statistics

## Abstract

*Background*: Metabolic Dysfunction-Associated Steatotic Liver Disease (MASLD), a novel term for Non-Alcoholic Fatty Liver Disease (NAFLD), is associated with liver mitochondrial dysfunction. We previously demonstrated that mitochondrial respiratory capacity in peripheral blood mononuclear cells (PBMCs) was significantly reduced in patients with MASLD compared to non-MASLD controls. For MASLD treatment, guidelines recommend behavioral and dietary changes to reduce body weight. A recent 12-month clinical trial demonstrated that ameliorating patients’ lifestyles through improved adherence to the Mediterranean diet and encouraged physical activity results in MASLD remission or regression. *Methods*: As a sub-study of the 12-month clinical trial, we evaluated the effects of the Mediterranean diet-oriented intervention on PBMC mitochondrial DNA content and respiratory parameters and on various biomarkers associated with MASLD. *Results*: Contrary to what was found at the baseline, after twelve months of intervention, systemic inflammatory and bioenergetics parameters did not differ between MASLD patients (N = 15) and control subjects (N = 17). PBMCs from MASLD subjects showed rescued basal respiration, ATP-linked respiration, maximal respiration, and spare respiratory capacity. The observed recovery coincided with a significant increase in the patients’ adherence to the Mediterranean diet (Medscore). *Conclusions:* Our findings indicate that a Mediterranean diet-oriented intervention, without calorie reduction, preserves blood cell mitochondrial function in MASLD subjects. Thus, PBMC bioenergetics-based assays might be taken into account not only for diagnosing but also for monitoring therapeutic responses in MASLD.

## 1. Introduction

Metabolic Dysfunction-Associated Steatotic Liver Disease (MASLD) [1], previously named Non-Alcoholic Fatty Liver Disease (NAFLD) [2,3], is the most common chronic liver disease. It consists of excessive triglyceride accumulation in the liver in the presence of at least one cardiometabolic risk factor in the absence of significant alcohol consumption among overweight or obesity, insulin resistance or type 2 diabetes, hypertriglyceridemia, low HDL cholesterol, and hypertension [1,3]. The new terms, Metabolic Dysfunction-Associated Fatty Liver Disease (MAFLD) [4] and Metabolic Dysfunction-Associated Steatotic Liver Disease (MASLD) [1,5], have been introduced to more accurately reflect this condition’s complex nature, which extends beyond mere hepatic steatosis [1,5]. While some debate surrounds the optimal definition of the disease, we have adopted the term MASLD in this work for several reasons. First, MASLD has been recommended as the preferred nomenclature following a multistep Delphi consensus process on fatty liver disease involving a wide range of global stakeholders [6]. Further, this terminology emphasizes the metabolic basis of the disease, which has long been recognized as the hepatic manifestation of metabolic syndrome. Importantly, MASLD avoids the stigmatizing label “fatty” [6]. Multiple re-analyses of existing cohort studies support that findings related to NAFLD can be fully extrapolated to individuals with MASLD [1].

The pathogenesis of MASLD is multifactorial, and mitochondrial dysfunction is a key contributor to its development and progression [7,8]. The study of blood-cell bioenergetics reflecting mitochondrial organ dysfunction has emerged for its applications in diagnostics [9]. We recently demonstrated that mitochondrial respiratory capacity was significantly reduced in peripheral blood mononuclear cells (PBMCs) from patients with MASLD compared to non-MASLD cases [10]. Thus, we proposed including mitochondrial respiratory parameters in panels of MASLD diagnostic biomarkers to monitor disease progression and possibly treatment response [10].

Due to the lack of specific therapies, nowadays, the principal recommendation for MASLD treatment consists of the adoption of a healthy lifestyle. Indeed, dietary and behavioral therapy-induced weight loss is strongly recommended in all patients with MASLD [1]. A holistic, person-centered care combining nutritional counseling and increased physical activity is required for disease management to favor steatosis regression and prevent complications [1,5]. Given the prevalence of the disease, lifestyle changes should be easily achievable by patients under the coordination of general practitioners or dieticians [3]. The Mediterranean Diet (MD) scheme is one of the most recommendable among the dietary interventions [1,11]. The MD, which is characterized by high consumption of vegetables, whole grains, legumes, nuts, and olive oil, with a moderate intake of fish and poultry and low consumption of red meat and sweets, has garnered significant attention for its potential protective effects against various metabolic disorders, including metabolic syndrome, type II diabetes, and MASLD [12,13,14]. While various studies showed that MD may improve anthropometric measures, lipid profile, glycemic indexes, liver enzymes, and MASLD severity indices [15], recent meta-analyses evidenced that, without combined exercise interventions, MD per se reduces intrahepatic lipid content but does not improve other circulating parameters among patients with MASLD [14]. We recently took part in a 12-month clinical trial that demonstrated that a semi-personalized intervention aimed to improve patient adherence to MD and motivate them to increase physical activity was easy to implement in clinical practice, improved some metabolic parameters, and effectively reduced liver steatosis in MASLD patients [16].

Of interest, emerging evidence suggests that dietary interventions, including MD, also ameliorate mitochondrial dysfunction [17]. Though the protective effects of the MD are the result of the whole dietary pattern rather than individual components, a body of preclinical studies demonstrated that some components of the MD (e.g., olive oil, plant-derived compounds rich in polyphenols, and polyunsaturated fatty acids) exert antioxidant properties and improve mitochondrial dynamics, biogenesis, and respiratory function [18,19].

In our previous paper [10], we reported the baseline data of a sub-study of the trial by Zanini and colleagues [16]. We measured real-time mitochondrial respirometry and observed a significant reduction in basal respiration, ATP-linked respiration, maximal respiration, and spare respiratory capacity in PBMCs from patients with MASLD compared to healthy controls [10]. Moreover, some mitochondrial respiratory parameters (ATP-production, basal respiration, spare respiratory capacity) were negatively correlated with anthropometric parameters, such as Body Mass Index (BMI) and waist circumference (WC), and with MASLD-known biomarkers, including fasting levels of insulin, glucose, total cholesterol, triglycerides, and levels of circulating inflammatory markers such as high-sensitivity C-reactive protein (hsCRP) and Interleukin-6 (IL-6) [10].

The same cohort of our study [10], with a net of 5 dropouts, concluded the 12-month longitudinal study [16]. Circulating biomarkers, PBMCs’ bioenergetics, and MD adherence score (Medscore) [16] were assessed at the study’s beginning (T0) and end (T12). Here, we show that twelve months of semi-personalized MD-oriented intervention can rescue PBMC mitochondrial respiratory parameters in subjects with MASLD.

## 2. Materials and Methods

### 2.1. Study Design and Participants

Patients with MASLD and voluntary control participants were enrolled to take part in a sub-study of the non-randomized clinical trial SEELN (from the Italian “Steatosi Epatica nonalcolica: Epidemiologia nutrizionale e Lifestyle mediciNe”). The main study received approval from the Ethical Committee of the Brescia District (code NP2587). It was registered on ClinicalTrials.gov as “Non-Alcoholic Fatty Liver Disease: Nutritional Epidemiology and Lifestyle Medicine” (code NCT03300661). The SEELN longitudinal trial aimed to evaluate the effect of twelve months of lifestyle guidance based on adherence to MD and exercise recommendations on MASLD-related outcomes. The exclusion criteria were the presence of severe medical conditions, being pregnant or breastfeeding throughout the study, and a history of liver disease or associated risk factors (heavy alcohol consumption, hepatitis virus infection, hemochromatosis, among others). Signing of informed consent was required for participation [16].

Mitochondrial respirometry parameters in PBMCs from both MASLD patients and non-MASLD control subjects were examined at the baseline in a subset of the SEELN study cohort, consisting of 37 participants aged 40–59 years, as previously described [10]. Bioenergetics analyses were then performed at the end of the study to evaluate changes after the MD-oriented intervention effects. Five subjects (4 MASLD cases and 1 non-MASLD case) dropped out during the study. Therefore, 32 subjects aged 40–59 years: 15 patients with MASLD (7 women and 8 men) and 17 non-MASLD controls (11 women and 6 men) were longitudinally examined. The two subgroups were matched for both age and sex. At the time of recruitment (T0) and again after 12 months (T12), all participants underwent the following procedures: (i) liver ultrasound (US), (ii) clinical and anthropometric assessments, (iii) blood collection for biochemical analysis and PBMC respirometry evaluation, and (iv) nutritional screening [16]. The diagnosis of MASLD was based on liver US, performed by an experienced gastroenterologist. Body Mass Index (BMI) was calculated by dividing the participants’ weight in kilograms by the square of their height in meters. The fatty liver index was calculated as described [20].

### 2.2. Nutritional Assessment and Intervention

A registered dietician collected dietary habits, assessed using a self-administered Italian semiquantitative Food Frequency Questionnaire (FFQ) previously developed and validated by our group [21]. The dietician also administered a Mediterranean Diet adherence questionnaire (Medscore), which ranges from 0 to 25 (with higher scores indicating better adherence). Our group developed this score based on a specific set of food items from the semiquantitative FFQ (Table S1) [16].

Our previous paper describes the Mediterranean diet-oriented intervention in detail [16]. The MASLD patients underwent a clinical evaluation at recruitment (T0) and every three months until the end of the 12-month intervention. During each clinical evaluation, weight, height, and waist circumference were assessed, and the dietician administered and evaluated the FFQ and Medscore questionnaires to encourage increased or decreased frequency consumption of healthy or unhealthy foods, respectively. The dietician provided three simple and tasty Mediterranean recipes at each time point. No calorie counting or restrictions were implemented. Although no specific questionnaire or tool was used to assess physical activity, the lifestyle intervention consisted of personalized suggestions to encourage exercise in daily life, according to patients’ preferences (for example, walking or cycling to work, brisk walking outdoors, swimming, etc.). Thus, agreeing on gradual and realistic objectives for a healthy diet and exercise and assisting with regular follow-up were adopted as a semi-personalized intervention. The control group received written general advice based on current Italian food dietary guidelines at recruitment (T0) and did not undergo physical examinations or dietary habits assessment every three months. Both groups were thoroughly evaluated at the end of the study (T12).

### 2.3. Measurement of Laboratory Data

Blood samples were obtained after an overnight fast using lithium–heparin tubes to collect plasma. These samples were then analyzed for glucose, total cholesterol, LDL cholesterol, HDL cholesterol, triglycerides, high-sensitivity C-reactive protein (hsCRP), alanine aminotransferase (ALT), aspartate aminotransferase (AST), gamma-glutamyl transferase (GGT), and total bilirubin. Fasting insulin and haptoglobin were detected in the serum. Commercially available assays were used according to the manufacturer’s instructions (Roche Diagnostics, Monza, Italy) on calibrated instruments verified using the registered quality controls. The Homeostasis Model Assessment (HOMA) index [22] was calculated as the product of the fasting plasma insulin level (µU/mL) and the fasting plasma glucose level (mmol/L) divided by 22.5.

### 2.4. Isolation of PBMCs

The isolation of PBCMs from venous blood was performed according to the Sepmate™ method (Stemcell Technologies, Vancouver, BC, Canada) with the Lymphoprep™ 1.077 g/mL density gradient medium (Stemcell Technologies). Briefly, the blood sample and an equal volume of Phosphate-Buffered Saline (PBS) (Immunological Science, Bolney, Sussex, UK) containing 2% Fetal Bovine Serum (FBS) (Immunological Science) were mixed and slowly layered on top of the Lymphoprep™ gradient. The tube was then spun at 1200× *g* for 10 min with the brake on. The PBMC layer was collected. PBMCs were washed twice with fresh PBS + 2% FBS medium by centrifuging at 300× *g* for 8 min. Cells were counted, and viability was checked with trypan blue staining using the TC20 automated cell counter (Bio-Rad Laboratories, Hercules, CA, USA) and immediately used for the Extracellular Flux Analysis. Residual PBMC pellets were stored at −80 °C for subsequent quantification of mitochondrial DNA (mtDNA).

### 2.5. Extracellular Flux Analysis of PBMCs

The Seahorse XFe24 Extracellular Flux Analyzer (Agilent, Santa Clara, CA, USA) was used to measure mitochondrial respirometry analysis of PBMCs, as previously described [10]. After PBMC isolation, cells were resuspended in Seahorse XF Base Medium (Agilent) containing 5.5 mM glucose, 2 mM L-glutamine, and 1 mM sodium pyruvate (with pH adjusted at 7.4). A total of 600,000 cells/well were seeded in quadruplicate in 200 μL/well on Seahorse XFe24 V7 PS Cell Culture Microplate (Agilent) pre-coated with Corning^®^ Cell-Tak™ cell and tissue adhesive (Corning, Discovery Labware, Bedford, MA, USA). Double centrifugation at 200× *g* for 1 min with the brake-off was performed to force adhesion, after which the medium volume was brought up to 525 µL in each well. Finally, microplates were incubated at 37 °C in a non-CO_2_ incubator for 1 h before running the assay. The Seahorse XF Cell Mito Stress Test (Agilent) was applied to measure cell oxygen consumption rate (OCR). Following the manufacturer’s instructions, the protocol was set to add mitochondrial modulators (75 µL/injection port) in sequential order: oligomycin (2 µM final concentration), carbonyl cyanide-4-(trifluoromethoxy), phenylhydrazone (FCCP, 1 µM), and a mixture of rotenone and antimycin A (both at 0.5 µM). The Wave software version 2.6.3 Agilent) was used to retrieve and automatically analyze data. Normalization was then carried out on DNA content with the CyQUANT Cell Proliferation Assay Kit (Thermo Fisher Scientific, Waltham, MA, USA), following the manufacturer’s protocol.

### 2.6. Mitochondrial DNA Quantification

Total DNA extraction and quantification of mtDNA content were performed as we previously described [10] by measuring the human gene of the mitochondrially encoded NADH dehydrogenase 1 (ND1) relative to the nuclear gene β-2 microglobulin (β2-m). A real-time quantitative PCR (qPCR) assay was performed on 20 ng DNA with the iTaq Universal SYBR Green Supermix (Bio-Rad Laboratories) on the ViiA7 Real-Time PCR system (Applied Biosystems, Waltham, MA, USA). Each sample reaction was conducted in triplicate. The following protocol was performed: initial “hot start” activation step at 95 °C for 5 min, followed by 40 cycles at 95 °C for 15 s and at 60 °C for 1 min using the ViiA7 instrument (Applied Biosystems). The following primer pairs were used (5′-3′): ND1 forward (GTCAACCTCGCTTCCCCACCCT) and reverse (TCCTGCGAATAGGCTTCCGGCT) for mtDNA and β2-m forward (CGACGGGAGGGTCGGGACAA) and reverse (GCCCCGCGAAAGAGCGGAAG) for nuclear DNA [23]. Mitochondrial DNA copy number was calculated as previously described [10] with the formula 2 × 2^−∆Ct^ (with ∆Ct = mtDNA Ct − nDNA Ct). Data were log-transformed to reduce skewness [24].

### 2.7. Measurement of Serum Cytokine IL-6 and TNF-α

Serum TNF-α and IL-6 levels were quantified using the Human TNF-alpha Quantikine HS ELISA (HSTA00E, R&D Systems, Minneapolis, MN, USA) and Human IL-6 Quantikine HS ELISA Kit (HS600C, R&D Systems), respectively, following the manufacturer’s instructions.

### 2.8. Statistical Analysis

Descriptive statistics were applied to variables of different natures. Specifically, for quantitative variables, the number of missing values (N-Miss), mean, Standard Deviation (SD), median, first quartile (Q1), third quartile (Q3), and range (minimum–maximum) were calculated. Frequencies were computed in absolute and percentage (%) terms for categorical variables. Data were stratified by diagnosis (MASLD vs. non-MASLD) to identify significant differences (*p*-value < 0.05) between these two subpopulations. The same approach was also applied when stratifying with respect to the time. The Wilcoxon Rank Sum test was computed in correspondence with quantitative variables, while Fisher’s exact test was used for categorical variables (*p*-values are reported in the Tables).

Mitochondrial respirometry variables, mtDNA copy number (mtDNAcn), and inflammatory parameters were z-scored before computing descriptive statistics. The variables analyzed are derived from non-diagnostic assays and subjected to inter-experimental variability. This simple approach permits comparing their means at two different time points (at T0 and T12).

Scatter dot plots were used to visualize the quantitative variables of the dataset. All these graphs were stratified with respect to diagnosis to compare patients with controls. The graphs also report median and interquartile range and corresponding Wilcoxon test *p*-values in correspondence of significantly different subpopulations (* *p*-values < 0.05; ** *p*-values < 0.01; *** *p*-values < 0.005; **** *p*-values < 0.001).

To evaluate dynamic changes in mitochondrial parameters of MASLD and non-MASLD over time, a linear mixed-effects model (LMM) was estimated using the Restricted Maximum Likelihood (REML) method, which is well suited for small sample sizes since it provides unbiased estimates of variance components. The corresponding *p*-values are computed by means of *t*-tests based on Satterthwaite’s approximation.

All analyses were computed using R 4.3.2. Scatter dot plots were performed using Prism 9.0.0 (GraphPad, Boston, MA, USA).

## 3. Results

### 3.1. Descriptive Statistics at Baseline (T0) and Follow-Up (T12)

#### 3.1.1. Adherence to Mediterranean Diet

**Figure 1:**
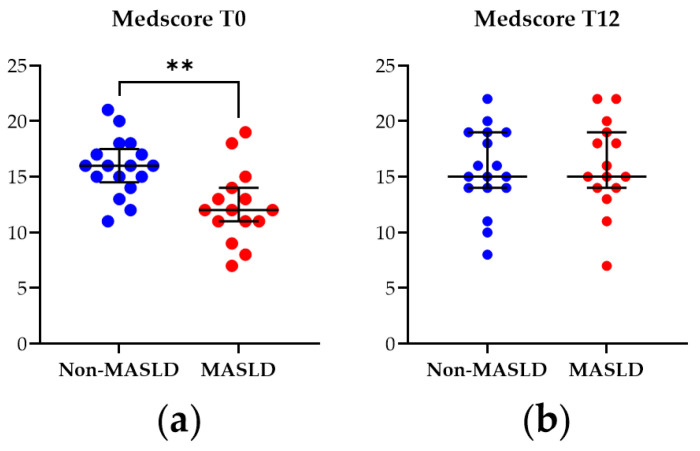
Mediterranean diet adherence score (Medscore) from patients with MASLD and non-MASLD controls at the baseline T0 (**a**) and at T12 (**b**). Data represent median ± interquartile range. Statistical analysis has been performed with Wilcoxon Rank Sum test. ** corresponds to *p*-value < 0.01. Graphs were performed using Prism 9.0.0 (GraphPad).

The Medscore was applied to assess adherence to the MD in the study cohort at baseline (T0) and after the 12-month nutritional intervention (T12). As illustrated in Figure 1, at T0, patients diagnosed with MASLD exhibited a significantly lower Medscore than non-MASLD controls (*p*-value = 0.003). However, by T12, this difference was no longer observed, indicating that the MASLD patients adhered to the dietician’s nutritional instructions and suggesting the intervention modality’s effectiveness.

#### 3.1.2. Clinical, Anthropometric, and Biochemical Characteristics of the Subjects

The baseline characteristics of the study cohort (32 subjects since 5 dropouts were excluded from the baseline), including MASLD and non-MASLD control cases diagnosed via liver US, are presented in Table S2. These findings are consistent with those reported in the previous study containing 37 subjects [10] and underline that, although the sample is small, even excluding its 13.51% (5) dropout, the results remain unchanged. Briefly, the MASLD group exhibited significantly higher waist circumference and BMI when compared to the control group (*p*-value < 0.001 for both variables). Furthermore, MASLD patients showed significantly higher levels of fasting glucose (*p*-value = 0.022) and insulin (*p*-value < 0.001) compared to controls. Consequently, the HOMA index was elevated in MASLD patients (*p*-value < 0.001), indicating insulin resistance. Triglycerides (*p*-value = 0.005) and LDL cholesterol (*p*-value = 0.030) levels were also higher in MASLD patients, while HDL cholesterol levels were lower (*p*-value = 0.008) compared to controls. Inflammatory markers, such as hsCRP (*p* = 0.009) and IL-6 (*p*-value = 0.022), were significantly higher in MASLD patients. However, the increase in TNF-α observed in MASLD cases from the previous cohort of 37 subjects was not significant (*p* = 0.063) in the current cohort of 32 subjects. Finally, the Fatty Liver Index was significantly higher in MASLD patients compared to non-MASLD controls (*p*-value < 0.001).

At T12, the situation changed (Table 1). The significant differences in HDL and LDL cholesterol levels observed at T0 between MASLD and non-MASLD patients were no longer present, indicating improved lipid metabolism. Additionally, the inflammatory state was reduced at T12 as the differences in circulating hsCRP and IL-6 levels observed at the baseline disappeared after the intervention (*p*-values = 0.072 and 0.984, respectively). However, a significant increase in TNF-α levels emerged in MASLD patients compared to controls (*p*-value = 0.016) in the smaller cohort.

#### 3.1.3. Mitochondrial DNA Content and Function in PBMCs from the Study Cohort

**Figure 2:**
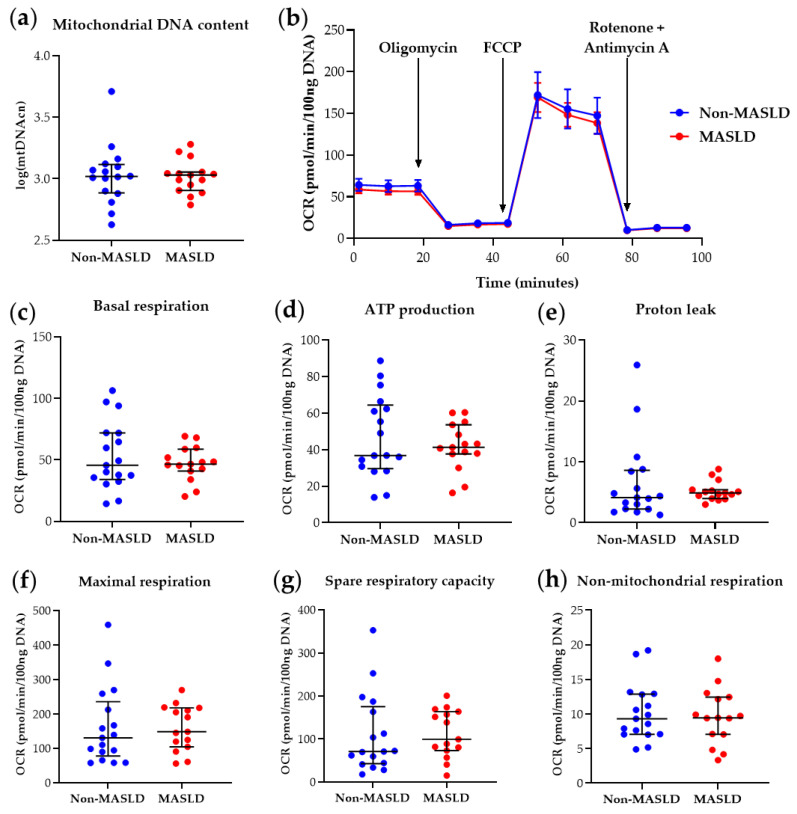
Mitochondrial DNA content and respiratory function in PBMCs from MASLD patients and non-MASLD controls at T12. (**a**) Quantification of mitochondrial DNA copy number (mtDNAcn) with values log-transformed. (**b**) Time-course representation of oxygen consumption rate (OCR) measured in live PBMCs during the Mito Stress Test. Three measurements for each condition were performed at different times by the Seahorse XFe24 analyzer. OCR values were normalized to DNA content. (**c**–**h**) Scatter dot plots of OCR values from individual subjects, normalized to DNA content, for each respiratory parameter. Data represent median ± interquartile range (**a**,**c**–**h**) or mean ± SEM (**b**). The Wilcoxon Rank Sum test was applied to compare the non-MASLD and MASLD subpopulations. Graphs were performed using Prism 9.0.0 (GraphPad).

The determination of PBMC mtDNA content and mitochondrial parameters derived from the Mito Stress Test in the 32-subject cohort at T0 confirmed the previous findings [10], showing reduced basal respiration (*p*-value = 0.025), maximal respiration (*p*-value = 0.043), ATP production-associated respiration (*p*-value = 0.030), and spare respiratory capacity (*p*-value = 0.047) in MASLD patients compared with controls (see Table S3 and Figure S1). At T12, as shown in Table S4 and Figure 2, MASLD subjects did not exhibit significant differences in PBMC mitochondrial respiratory parameters compared to the non-MASLD cohort. These results indicate that the intervention ameliorated blood cell bioenergetics in MASLD patients, with the significant differences observed at T0 no longer present (Table S4 and Figure 2).

The same type of analysis was applied to the clinical, anthropometric, and biochemical variables to compare values at baseline (T0) versus the final follow-up (T12) in the two subgroups of MASLD patients and controls, without any significant result. Moreover, for evaluating the dynamic changes of mitochondrial parameters over time, an LMM was estimated, and the results are reported in Table S5. It contains the coefficients of the model that reflect the average differences in each parameter computed over time. Positive values suggest that, on average, the parameters tend to increase, whereas negative values indicate a decrease. Since all *p*-values exceed 0.05, these changes are not statistically significant, implying that there is no evidence that the mitochondrial parameters vary meaningfully between baseline and T12-month follow-up. However, this analysis should be interpreted with caution due to the limited sample size, which affects the statistical power of the model.

Figure 2 visualizes mitochondrial bioenergetics parameter data. Figure 2b illustrates the real-time PBMC Oxygen Consumption Rate (OCR) pattern measured during the Mito Stress Test after adding specific mitochondrial inhibitors. Figure 2c–h present scatter dot plots of individual data points for each respirometry parameter, automatically retrieved by Wave software, version 2.6.3. These results corroborate the involvement of mitochondrial impairment in MASLD, as demonstrated by various research groups in liver studies [7,8], and suggest that blood cell mitochondrial dysfunction, possibly reflecting liver and/or systemic mitochondrial derangements, can be rescued by therapeutic intervention(s). Furthermore, the restoration of basal respiration, ATP production-associated respiration, maximal respiration, and spare respiratory capacity at T12 in circulating PBMCs from MASLD patients who received reinforced nutritional and lifestyle advice suggests that measuring key respiratory parameters in blood cells is helpful for therapy monitoring.

## 4. Discussion

The semi-personalized intervention based on MD-oriented modification of dietary patterns and lifestyle counseling described in the present paper has recently been demonstrated as efficacious in reducing liver steatosis in a larger cohort of patients with MASLD [16]. Steatosis severity was downgraded in almost half of the patients, and steatosis remission was achieved in about 20% of cases, mostly presenting milder steatosis at baseline [16]. As observed in the larger cohort, in the present sub-study, the Medscore values were lower in MASLD patients than in the healthy subjects at the baseline. Still, we confirmed that they were not significantly different at the end of the intervention, indicating an improvement only among MASLD patients who received the semi-personalized advice.

Since liver mitochondrial derangements play a major role in MASLD pathogenesis and progression [7,8], the main aim of the present study was to investigate possible changes in PBMC mitochondrial bioenergetics at the end of the 12-month intervention. We found that the MD-oriented approach rescued impaired mitochondrial respiration in PBMCs from patients with MASLD. Indeed, the significant differences between patients with MASLD and control subjects regarding mitochondrial respiratory parameters (including basal respiration, ATP production, maximal respiration, and spare respiratory capacity) found at baseline (T0) were no longer evident at the end of the study (T12).

The possible mechanisms underlying the healthy changes of mitochondrial parameters in patients with MASLD following a simple semi-personalized intervention, mainly based on improved adherence to MD, deserve to be discussed. We found that the intervention ameliorated established markers of metabolic disease (including HDL and LDL cholesterol) in the sub-study MASLD cohort. In particular, the significant reduction in HDL cholesterol levels observed in MASLD patients compared to non-MASLD controls at T0 was no longer present at T12. Notably, using Spearman analysis, we previously demonstrated a significant positive correlation between HDL cholesterol levels and most PBMC mitochondrial respiratory parameters (basal respiration, ATP production, maximal respiration, and spare respiratory capacity) [10]. Whether normalization of HDL cholesterol levels could partly explain the improved mitochondrial bioenergetics deserves further investigation, especially considering the protective role of HDL cholesterol against MASLD-related cardiovascular disease risk [25].

Of particular interest, values of circulating biomarkers indicative of inflammatory status (i.e., hsCRP and IL-6) known to be correlated with MASLD [26] were significantly higher in MASLD vs. non-MASLD cases at the baseline but comparable in the two cohorts at the end of the study. Interleukin-6 released by inflamed adipose tissue is mainly responsible for increased blood CRP concentration and highly contributes to hepatic inflammation [5]. The analysis of TNF-α levels gave unexpected results in our cohort, a point to be further addressed in larger samples. In line with other reports [27], increased IL-6 showed a strong negative correlation with PBMC maximal respiration and spare respiratory capacity in our previous study [10]. Notably, aerobic and resistance exercise, known to be beneficial in MASLD management, reduces circulating IL-6 levels [5]. The role of reduced HDL cholesterol and increased IL-6 and hsCRP levels might also be interconnected since subjects with low plasma HDL cholesterol display increased inflammatory biomarkers, including CRP [28]. Our observations suggest that a combined intervention mainly based on improved MD adherence and encouraging patients to practice physical activity can reduce some of the main drivers of MASLD progression toward more severe conditions [3].

There are several limitations in the present study. The small sample size limited our ability to draw definitive conclusions regarding possible mechanisms accounting for the recovery of mitochondrial function. Though the Seahorse Extracellular Flux Analyzer technique is not currently validated for routine, it offers several advantages over other methods for assessing mitochondrial respiration, which have lower sensitivity and require more blood-derived cells [9]. Using freshly isolated PBMC guarantees excellent respirometry measures but has some disadvantages since it limits comparative longitudinal studies, which renders necessary z-scoring of results. Most recently, novel methodologies of PBMC cryopreservation for bioenergetics analyses have been described [29], and we have set up a reliable method to use previously isolated and cryopreserved PBMCs for future longitudinal studies.

## 5. Conclusions

Our findings indicate that blood cell mitochondrial bioenergetics might be included in the MASLD diagnostic panels to monitor disease progression or therapeutic response. Further, our observations suggest that adhering to a newly proposed MD-oriented intervention, which is practical and easy to achieve in real-world settings, positively impacts blood cell mitochondrial bioenergetics as an effective approach to MASLD treatment.

## Figures and Tables

**Table 1:** Descriptive statistics on clinical variables and analytes of the study cohorts at follow-up (T12).

Variables	MASLD (N = 15)	Non-MASLD (N = 17)	Total (N = 32)	*p* Value
**Steatosis**				***<0.001*** ^(a)^
Absent	4 (26.7%)	17 (100.0%)	21 (65.6%)	
Present	11 (73.3%)	0 (0.0%)	11 (34.4%)	
**Sex**				0.476 ^(a)^
Female	7 (46.7%)	11 (64.7%)	18 (56.2%)	
Male	8 (53.3%)	6 (35.3%)	14 (43.8%)	
**Age (years)**				0.130 ^(b)^
Mean (SD)	52.40 (4.48)	49.41 (5.91)	50.81 (5.42)	
Median (Q1, Q3)	53.00 (48.50, 55.50)	50.00 (44.00, 53.00)	51.50 (46.50, 55.00)	
Range	44.00–60.00	41.00–60.00	41.00–60.00	
**Medscore**				0.864 ^(b)^
Mean (SD)	15.93 (4.06)	15.59 (3.71)	15.75 (3.82)	
Median (Q1, Q3)	15.00 (14.00, 18.50)	15.00 (14.00, 19.00)	15.00 (14.00, 19.00)	
Range	7.00–22.00	8.00–22.00	7.00–22.00	
**BMI (Kg/m^2^)**				***<0.001*** ^(b)^
Mean (SD)	31.27 (8.09)	23.57 (3.61)	27.18 (7.17)	
Median (Q1, Q3)	30.70 (26.05, 33.80)	24.10 (20.60, 26.20)	26.05 (23.43, 30.18)	
Range	22.90–55.70	17.20–30.00	17.20–55.70	
**Waist circumference (cm**)				***0.001*** ^(b)^
Mean (SD)	105.37 (17.62)	87.96 (10.04)	96.12 (16.44)	
Median (Q1, Q3)	103.00 (94.50, 110.25)	89.00 (82.00, 97.00)	95.50 (85.50, 103.00)	
Range	84.00–160.00	71.00–102.00	71.00–160.00	
**Fasting glucose (mg/dL)**				***0.028*** ^(b)^
Mean (SD)	97.16 (12.60)	86.24 (14.50)	91.36 (14.52)	
Median (Q1, Q3)	96.00 (88.00, 105.50)	82.00 (77.00, 100.27)	91.50 (80.92, 102.28)	
Range	76.00–123.34	56.54–107.90	56.54–123.34	
**Fasting insulin (µUI/mL)**				***0.002*** ^(b)^
Mean (SD)	14.35 (11.02)	4.48 (3.56)	9.11 (9.29)	
Median (Q1, Q3)	12.00 (7.50, 18.50)	4.00 (1.00, 6.00)	6.50 (2.75, 12.25)	
Range	1.00–38.00	1.00–13.00	1.00–38.00	
**HOMA index**				***0.002*** ^(b)^
Mean (SD)	3.48 (2.64)	1.01 (0.94)	2.17 (2.27)	
Median (Q1, Q3)	3.21 (1.56, 4.97)	0.73 (0.26, 1.38)	1.40 (0.51, 3.24)	
Range	0.21–9.29	0.19–3.43	0.19–9.29	
**Total cholesterol (mg/dL)**				0.282 ^(b)^
Mean (SD)	211.77 (34.04)	198.54 (32.81)	204.74 (33.53)	
Median (Q1, Q3)	215.00 (189.50, 228.00)	196.10 (185.00, 211.00)	202.80 (187.55, 226.50)	
Range	147.00–267.00	128.00–254.70	128.00–267.00	
**HDL cholesterol (mg/dL)**				0.062 ^(b)^
Mean (SD)	55.99 (16.20)	65.75 (12.34)	61.17 (14.89)	
Median (Q1, Q3)	57.80 (40.55, 68.65)	68.20 (56.10, 75.00)	64.90 (47.77, 74.80)	
Range	36.60–85.60	44.80–84.20	36.60–85.60	
**LDL cholesterol (mg/dL)**				0.117 ^(b)^
Mean (SD)	131.45 (29.01)	114.85 (34.09)	122.63 (32.42)	
Median (Q1, Q3)	141.00 (116.50, 149.50)	116.00 (92.60, 134.87)	121.00 (103.40, 148.25)	
Range	74.00–169.00	46.00–185.51	46.00–185.51	
**Triglycerides (mg/dL)**				***0.019*** ^(b)^
Mean (SD)	128.80 (65.73)	86.12 (49.49)	106.12 (60.69)	
Median (Q1, Q3)	108.00 (72.00, 158.50)	76.00 (55.00, 91.00)	82.00 (62.00, 135.25)	
Range	62.00–270.00	37.00–206.00	37.00–270.00	
**ALT (U/L)**				0.734 ^(b)^
Mean (SD)	29.41 (12.08)	28.40 (12.69)	28.87 (12.22)	
Median (Q1, Q3)	26.00 (21.15, 37.90)	25.00 (23.00, 31.00)	26.00 (21.98, 31.33)	
Range	12.00–54.00	17.00–73.10	12.00–73.10	
**AST (U/L)**				0.471 ^(b)^
Mean (SD)	16.05 (3.38)	15.90 (5.18)	15.97 (4.36)	
Median (Q1, Q3)	16.00 (14.00, 18.50)	14.00 (12.00, 20.00)	14.75 (13.00, 19.00)	
Range	10.00–22.00	10.00–27.10	10.00–27.10	
**Total bilirubin (mg/dL)**				0.677 ^(b)^
N-Miss	1	0	1	
Mean (SD)	0.43 (0.11)	0.53 (0.37)	0.48 (0.29)	
Median (Q1, Q3)	0.41 (0.36, 0.50)	0.48 (0.35, 0.53)	0.42 (0.35, 0.51)	
Range	0.26–0.62	0.18–1.83	0.18–1.83	
**GGT (U/L)**				0.751 ^(b)^
N-Miss	1	0	1	
Mean (SD)	35.82 (37.35)	27.24 (22.43)	31.11 (29.86)	
Median (Q1, Q3)	21.80 (13.47, 42.25)	19.30 (10.40, 33.50)	21.40 (11.50, 34.70)	
Range	6.90–144.70	7.90–86.90	6.90–144.70	
**Haptoglobin (mg/dL)**				0.092 ^(b)^
N-Miss	1	0	1	
Mean (SD)	134.21 (58.55)	97.65 (41.50)	114.16 (52.40)	
Median (Q1, Q3)	126.50 (101.00, 147.00)	103.00 (69.00, 123.00)	117.00 (86.00, 136.00)	
Range	28.00–260.00	21.00–169.00	21.00–260.00	
**hsCRP**				0.072 ^(b)^
N-Miss	1	4	5	
Mean (SD)	0.28 (1.28)	−0.30 (0.47)	0.00 (1.00)	
Median (Q1, Q3)	−0.29 (−0.43, −0.04)	−0.40 (−0.54, −0.31)	−0.32 (−0.50, −0.20)	
Range	−0.51–3.10	−0.59–1.20	−0.59–3.10	
**TNF** **-α**				***0.016*** ^(b)^
N-Miss	0	1	1	
Mean (SD)	0.45 (1.02)	−0.42 (0.80)	0.00 (1.00)	
Median (Q1, Q3)	0.32 (−0.12, 1.30)	−0.75 (−0.96, 0.13)	−0.05 (−0.81, 0.80)	
Range	−1.55–2.12	−1.58–0.85	−1.58–2.12	
**IL-6**				0.984 ^(b)^
N-Miss	0	1	1	
Mean (SD)	0.01 (0.99)	−0.01 (1.04)	0.00 (1.00)	
Median (Q1, Q3)	−0.29 (−0.74, 0.34)	−0.48 (−0.74, 0.80)	−0.29 (−0.74, 0.68)	
Range	−0.97–2.83	−1.17–2.83	−1.17–2.83	
**Fatty Liver Index**				***0.001*** ^(b)^
N-Miss	1	0	1	
Mean (SD)	64.82 (26.88)	26.89 (22.46)	44.02 (30.83)	
Median (Q1, Q3)	73.85 (43.15, 85.77)	22.28 (4.91, 39.38)	39.38 (17.68, 73.85)	
Range	14.59–99.85	1.32–79.73	1.32–99.85	

^(a)^ Fisher exact test; ^(b)^ Wilcoxon Rank Sum Test. In bold and italics, *p*-values < 0.05. Acronyms stand for: ALT, Alanine aminotransferase; AST, Aspartate aminotransferase; BMI, Body Mass Index; GGT, Gamma-Glutamyl Transferase; HOMA, Homeostasis Model Assessment; hsCRP, high-sensitivity C-Reactive Protein; IL-6, Interleukin-6; TNF-α, Tumor Necrosis Factor-α.

## Data Availability

The data presented in this study are available upon request from the corresponding author. Due to privacy restrictions, they are not publicly available.

## Supplementary Materials

The following supporting information can be downloaded at: https://www.mdpi.com/article/10.3390/diagnostics14182041/s1. Table S1: Criteria for score assignment in Medscore, according to selected food frequency consumption; Table S2: Descriptive statistics on clinical variables and analytes of the study cohorts at the baseline (T0); Table S3: Descriptive statistics on mitochondrial parameters at the baseline (T0); Table S4. Descriptive statistics on mitochondrial parameters at follow-up (T12); Table S5. Coefficients of a linear mixed-effect model on mitochondrial parameters evaluated over time. Figure S1: Mitochondrial content and function in PBMCs from NAFLD patients and non-NAFLD controls at the baseline (T0).

## References

[1] Tacke F., Horn P., Wai-Sun Wong V., Ratziu V., Bugianesi E., Francque S., Zelber-Sagi S., Valenti L., Roden M., Schick F. (2024). EASL–EASD–EASO Clinical Practice Guidelines on the Management of Metabolic Dysfunction-Associated Steatotic Liver Disease (MASLD). J. Hepatol..

[2] European Association for the Study of the Liver (EASL), European Association for the Study of Diabetes (EASD), European Association for the Study of Obesity (EASO) (2016). EASL–EASD–EASO Clinical Practice Guidelines for the Management of Non-Alcoholic Fatty Liver Disease. J. Hepatol..

[3] Powell E.E., Wong V.W.S., Rinella M. (2021). Non-Alcoholic Fatty Liver Disease. Lancet.

[4] Eslam M., Sanyal A.J., George J., Sanyal A., Neuschwander-Tetri B., Tiribelli C., Kleiner D.E., Brunt E., Bugianesi E., Yki-Järvinen H. (2020). MAFLD: A Consensus-Driven Proposed Nomenclature for Metabolic Associated Fatty Liver Disease. Gastroenterology.

[5] Targher G., Tilg H., Byrne C.D. (2021). Non-Alcoholic Fatty Liver Disease: A Multisystem Disease Requiring a Multidisciplinary and Holistic Approach. Lancet Gastroenterol. Hepatol..

[6] Rinella M.E., Lazarus J.V., Ratziu V., Francque S.M., Sanyal A.J., Kanwal F., Romero D., Abdelmalek M.F., Anstee Q.M., Arab J.P. (2023). A Multisociety Delphi Consensus Statement on New Fatty Liver Disease Nomenclature. J. Hepatol..

[7] Legaki A.-I., Moustakas I.I., Sikorska M., Papadopoulos G., Velliou R.-I., Chatzigeorgiou A. (2022). Hepatocyte Mitochondrial Dynamics and Bioenergetics in Obesity-Related Non-Alcoholic Fatty Liver Disease. Curr. Obes. Rep..

[8] Prasun P., Ginevic I., Oishi K. (2021). Mitochondrial Dysfunction in Nonalcoholic Fatty Liver Disease and Alcohol Related Liver Disease. Transl. Gastroenterol. Hepatol..

[9] Braganza A., Annarapu G.K., Shiva S. (2020). Blood-Based Bioenergetics: An Emerging Translational and Clinical Tool. Mol. Asp. Med..

[10] Garrafa E., Segala A., Vezzoli M., Bottani E., Zanini B., Vetturi A., Bracale R., Ricci C., Valerio A. (2023). Mitochondrial Dysfunction in Peripheral Blood Mononuclear Cells as Novel Diagnostic Tools for Non-Alcoholic Fatty Liver Disease: Visualizing Relationships with Known and Potential Disease Biomarkers. Diagnostics.

[11] Yannakoulia M., Scarmeas N. (2024). Diets. N. Engl. J. Med..

[12] Willett M.C. (2021). The Mediterranean Diet and Health: A Comprehensive Overview. Publ. J. Intern. Med..

[13] Martín-Peláez S., Fito M., Castaner O. (2020). Mediterranean Diet Effects on Type 2 Diabetes Prevention, Disease Progression, and Related Mechanisms. A Review. Nutrients.

[14] Houttu V., Csader S., Nieuwdorp M., Holleboom A.G., Schwab U. (2021). Dietary Interventions in Patients With Non-Alcoholic Fatty Liver Disease: A Systematic Review and Meta-Analysis. Front. Nutr..

[15] Moosavian S.P., Arab A., Paknahad Z. (2020). The Effect of a Mediterranean Diet on Metabolic Parameters in Patients with Non-Alcoholic Fatty Liver Disease: A Systematic Review of Randomized Controlled Trials. Clin. Nutr. ESPEN.

[16] Zanini B., Benini F., Marullo M., Simonetto A., Rossi A., Cavagnoli P., Bonalumi A., Marconi S., Pigozzi M.G., Gilioli G. (2024). Mediterranean-Oriented Dietary Intervention Is Effective to Reduce Liver Steatosis in Patients with Nonalcoholic Fatty Liver Disease: Results from an Italian Clinical Trial. Int. J. Clin. Pract..

[17] Kyriazis I., Vassi E., Alvanou M., Angelakis C., Skaperda Z., Tekos F., Garikipati V., Spandidos D., Kouretas D. (2022). The Impact of Diet upon Mitochondrial Physiology (Review). Int. J. Mol. Med..

[18] Khalil M., Shanmugam H., Abdallah H., John Britto J.S., Galerati I., Gómez-Ambrosi J., Frühbeck G., Portincasa P. (2022). The Potential of the Mediterranean Diet to Improve Mitochondrial Function in Experimental Models of Obesity and Metabolic Syndrome. Nutrients.

[19] Ruocco C., Ragni M., Tedesco L., Segala A., Servili M., Riccardi G., Carruba M.O., Valerio A., Nisoli E., Visioli F. (2022). Molecular and Metabolic Effects of Extra-Virgin Olive Oil on the Cardiovascular Gene Signature in Rodents. Nutr. Metab. Cardiovasc. Dis..

[20] Bedogni G., Bellentani S., Miglioli L., Masutti F., Passalacqua M., Castiglione A., Tiribelli C. (2006). The Fatty Liver Index: A Simple and Accurate Predictor of Hepatic Steatosis in the General Population. BMC Gastroenterol..

[21] Zanini B., Simonetto A., Bertolotti P., Marullo M., Marconi S., Becchetti C., Gilioli G., Valerio A., Donato F., Ricci C. (2020). A New Self-Administered Semi-Quantitative Food Frequency Questionnaire to Estimate Nutrient Intake among Italian Adults: Development Design and Validation Process. Nutr. Res..

[22] Matthews D.R., Hosker J.R., Rudenski A.S., Naylor B.A., Treacher D.F., Turner R.C. (1985). Homeostasis Model Assessment: Insulin Resistance and Fl-Cell Function from Fasting Plasma Glucose and Insulin Concentrations in Man. Diabetologia.

[23] Bonnen P.E., Yarham J.W., Besse A., Wu P., Faqeih E.A., Al-Asmari A.M., Saleh M.A.M., Eyaid W., Hadeel A., He L. (2013). Mutations in FBXL4 Cause Mitochondrial Encephalopathy and a Disorder of Mitochondrial DNA Maintenance. Am. J. Hum. Genet..

[24] Picard M., Prather A.A., Puterman E., Cuillerier A., Coccia M., Aschbacher K., Burelle Y., Epel E.S. (2018). A Mitochondrial Health Index Sensitive to Mood and Caregiving Stress. Biol. Psychiatry.

[25] Mocciaro G., Allison M., Jenkins B., Azzu V., Huang-Doran I., Herrera-Marcos L.V., Hall Z., Murgia A., Susan D., Frontini M. (2023). Non-Alcoholic Fatty Liver Disease Is Characterised by a Reduced Polyunsaturated Fatty Acid Transport via Free Fatty Acids and High-Density Lipoproteins (HDL). Mol. Metab..

[26] Duan Y., Pan X., Luo J., Xiao X., Li J., Bestman P.L., Luo M. (2022). Association of Inflammatory Cytokines With Non-Alcoholic Fatty Liver Disease. Front. Immunol..

[27] Tyrrell D.J., Bharadwaj M.S., Van Horn C.G., Marsh A.P., Nicklas B.J., Molina A.J.A. (2015). Blood-Cell Bioenergetics Are Associated with Physical Function and Inflammation in Overweight/Obese Older Adults. Exp. Gerontol..

[28] Holven K.B., Retterstøl K., Ueland T., Ulven S.M., Nenseter M.S., Sandvik M., Narverud I., Berge K.E., Ose L., Aukrust P. (2013). Subjects with Low Plasma HDL Cholesterol Levels Are Characterized by an Inflammatory and Oxidative Phenotype. PLoS ONE.

[29] Iwata K., Xie M.-J., Guest P.C., Hirai T., Matsuzazki H. (2022). Measurement of Mitochondrial Respiration in Cryopreserved Human Peripheral Blood Mononuclear Cells (PBMCs). Multiplex Biomarker Techniques: Methods and Applications for COVID-19 Disease Diagnosis and Risk Stratification.

