# Supplementary material for: A Mediterranean Diet-Oriented Intervention Rescues Impaired Blood Cell Bioenergetics in Patients with Metabolic Dysfunction-Associated Steatotic Liver Disease
Source: Diagnostics (Basel). 2024 Sep 14;14(18):2041. doi: 10.3390/diagnostics14182041 (PMC11431693; doi:10.3390/diagnostics14182041)
Supplement: Supplementary file 1 [file diagnostics-14-02041-s001.zip › diagnostics-3132519-supplementary.pdf]

A Mediterranean diet-oriented intervention rescues impaired blood cell bioenergetics in patients with Metabolic Dysfunction-Associated Steatotic Liver Disease

Agnese Segala et al.

Supplementary data

Table S1. Criteria for score assignment in Medscore, according to selected food frequency consumption

Food	Assigned score		
	0	1	2
Whole pasta	Less than 50% of the pasta consumption	More than 50% of the pasta consumption	/
Whole bread	<3 times /week	≥ 3 times/week	/
Vegetables	<6 times/week	6 times/week	>6 times/week
Fruits	<5 times/week	5 times/week	>5 times/week
Pulses	<1 time/week	1 time/week	>1 time/week
Fish	<1 time/week	≥ 1 time/week	/
Nuts	<1 time/week	≥1 and <7 times/week	≥7 times/week
Olive oil	<7 times/week	7 times/week	>7 times/week
Wine	>7 times/week	≤7 times/week	/
Processed meat	>1 time/week	≤1 time/week	/
Red meat	>1 time/week	≤1 time/week	/
Poultry and eggs	≥5 times/week	<5 times /week	
Sugary drinks	occasionally or more	never	/
Confectionery	>1 time/week	≤1 time/week	/
Cheese	>3 times/week	≤3 times/week	/
Yogurt	≤1 or >7 times/week	>1 and ≤ 7 times/week	/
Butter	≥ 3 times/week	<3 times /week	/
Spirits	>1 time/week	≤1 time/week	/
Water	<3 glasses/day	≥3 and <6 glasses/day	≥6 glasses/day

Table S2. Descriptive statistics on clinical variables and analytes of the study cohorts at the baseline (T0)

Variables	MASLD (N=15)	Non-MASLD (N=17)	Total (N=32)	p value
**Sex**				0.476 [(a)]
Female	7 (46.7%)	11 (64.7%)	18 (56.2%)	
Male	8 (53.3%)	6 (35.3%)	14 (43.8%)	
**Age (years)**				0.082 [(b)]
Mean (SD)	51.60 (4.47)	48.18 (5.80)	49.78 (5.42)	
Median (Q1, Q3)	52.00 (49.00, 54.50)	47.00 (43.00, 52.00)	50.50 (45.75, 54.00)	
Range	42.00 - 59.00	40.00 - 59.00	40.00 - 59.00	
**Medscore**				***0.003*** [(b)]
Mean (SD)	12.33 (3.29)	15.88 (2.60)	14.22 (3.41)	
Median (Q1, Q3)	12.00 (11.00, 13.50)	16.00 (15.00, 17.00)	14.50 (12.00, 16.25)	
Range	7.00 - 19.00	11.00 - 21.00	7.00 - 21.00	
**BMI (Kg/m^2)**				***< 0.001*** [(b)]
Mean (SD)	32.71 (7.42)	23.42 (3.73)	27.77 (7.36)	
Median (Q1, Q3)	31.30 (27.85, 34.55)	23.30 (20.30, 25.90)	26.70 (23.20, 31.35)	
Range	25.20 - 55.00	17.60 - 31.70	17.60 - 55.00	
**Waist circumference (cm)**				***< 0.001*** [(b)]
N-Miss	0	2	2	
Mean (SD)	108.13 (19.72)	86.43 (9.50)	97.28 (18.79)	
Median (Q1, Q3)	105.00 (96.50, 112.00)	83.00 (81.00, 94.25)	95.50 (83.75, 104.25)	
Range	86.00 - 169.00	70.00 - 99.00	70.00 - 169.00	
**Fasting glucose (mg/dL)**				***0.022*** [(b)]
Mean (SD)	101.73 (24.67)	86.94 (11.35)	93.88 (19.94)	
Median (Q1, Q3)	93.00 (85.00, 106.50)	82.00 (80.00, 96.00)	86.00 (81.00, 98.75)	
Range	81.00 - 169.00	74.00 - 115.00	74.00 - 169.00	
**Fasting insulin (µUI/mL)**				***< 0.001*** [(b)]
Mean (SD)	14.60 (10.76)	4.41 (3.48)	9.19 (9.23)	
Median (Q1, Q3)	11.00 (8.50, 18.00)	4.00 (2.00, 5.00)	7.00 (3.00, 11.25)	
Range	2.00 - 42.00	1.00 - 14.00	1.00 - 42.00	
**HOMA index**				***< 0.001*** [(b)]
Mean (SD)	3.79 (3.06)	1.02 (0.98)	2.32 (2.59)	
Median (Q1, Q3)	3.11 (1.72, 4.92)	0.79 (0.40, 1.21)	1.49 (0.62, 3.11)	
Range	0.42 - 11.27	0.18 - 3.98	0.18 - 11.27	
**Total cholesterol (mg/dL)**				0.050 [(b)]
Mean (SD)	209.87 (29.50)	186.88 (31.78)	197.66 (32.41)	
Median (Q1, Q3)	212.00 (189.50, 230.00)	185.00 (167.00, 202.00)	194.00 (178.50, 218.25)	
Range	165.00 - 262.00	117.00 - 259.00	117.00 - 262.00	
**HDL cholesterol (mg/dL)**				***0.008*** [(b)]
Mean (SD)	51.59 (14.86)	67.79 (17.43)	60.20 (18.00)	
Median (Q1, Q3)	47.60 (39.45, 62.80)	69.50 (52.90, 75.50)	56.95 (47.23, 73.20)	
Range	33.20 - 77.60	43.30 - 112.80	33.20 - 112.80	
**LDL cholesterol (mg/dL)**				***0.030*** [(b)]
Mean (SD)	130.13 (31.84)	103.29 (32.88)	115.88 (34.66)	
Median (Q1, Q3)	127.00 (110.00, 154.50)	97.00 (85.00, 121.00)	114.50 (93.00, 143.25)	
Range	72.00 - 194.00	37.00 - 166.00	37.00 - 194.00	

Variables	MASLD (N=15)	Non-MASLD (N=17)	Total (N=32)	p value
**Triglycerides (mg/dL)**				***0.005*** [(b)]
Mean (SD)	140.80 (78.64)	79.06 (46.65)	108.00 (69.97)	
Median (Q1, Q3)	126.00 (73.50, 184.00)	61.00 (49.00, 92.00)	83.00 (60.00, 131.75)	
Range	60.00 - 301.00	32.00 - 219.00	32.00 - 301.00	
**ALT (U/L)**				0.113 [(b)]
N-Miss	0	1	1	
Mean (SD)	35.07 (15.95)	26.31 (12.87)	30.55 (14.87)	
Median (Q1, Q3)	34.00 (22.50, 46.00)	21.50 (17.50, 31.25)	27.00 (19.00, 36.00)	
Range	13.00 - 64.00	15.00 - 58.00	13.00 - 64.00	
**AST (U/L)**				0.316 [(b)]
Mean (SD)	18.33 (6.08)	16.94 (9.48)	17.59 (7.98)	
Median (Q1, Q3)	19.00 (13.50, 22.50)	14.00 (10.00, 22.00)	17.00 (11.00, 22.25)	
Range	8.00 - 27.00	6.00 - 43.00	6.00 - 43.00	
**Total bilirubin (mg/dL)**				0.664 [(b)]
Mean (SD)	1.57 (3.86)	0.66 (0.29)	1.09 (2.64)	
Median (Q1, Q3)	0.55 (0.47, 0.70)	0.56 (0.53, 0.71)	0.56 (0.49, 0.72)	
Range	0.29 - 15.50	0.35 - 1.42	0.29 - 15.50	
**GGT (U/L)**				0.610 [(b)]
Mean (SD)	33.85 (45.73)	20.52 (15.93)	26.77 (33.48)	
Median (Q1, Q3)	18.00 (11.60, 27.65)	16.20 (12.60, 20.20)	16.40 (12.35, 26.08)	
Range	0.50 - 178.60	5.30 - 69.70	0.50 - 178.60	
**Haptoglobin (mg/dL)**				0.162 [(b)]
Mean (SD)	131.50 (60.31)	108.01 (50.11)	119.02 (55.50)	
Median (Q1, Q3)	141.00 (87.30, 170.00)	120.00 (65.00, 135.00)	121.00 (78.90, 160.50)	
Range	21.00 - 224.00	21.00 - 202.00	21.00 - 224.00	
**hsCRP**				***0.009*** [(b)]
Mean (SD)	0.38 (1.24)	-0.33 (0.58)	-0.00 (1.00)	
Median (Q1, Q3)	-0.29 (-0.46, 0.79)	-0.62 (-0.71, -0.16)	-0.46 (-0.63, 0.22)	
Range	-0.71 - 2.98	-0.80 - 1.06	-0.80 - 2.98	
**TNF-α**				0.063 [(b)]
N-Miss	0	1	1	
Mean (SD)	0.28 (1.12)	-0.27 (0.82)	-0.00 (1.00)	
Median (Q1, Q3)	-0.04 (-0.22, 0.36)	-0.29 (-0.83, 0.04)	-0.13 (-0.50, 0.36)	
Range	-0.70 - 3.98	-1.68 - 1.76	-1.68 - 3.98	
**IL-6**				***0.022*** [(b)]
N-Miss	0.38 (1.29)	-0.33 (0.47)	0.00 (1.00)	
Mean (SD)	-0.04 (-0.35, 0.53)	-0.44 (-0.63, -0.16)	-0.31 (-0.51, -0.03)	
Range	-0.66 - 4.22	-0.84 - 1.27	-0.84 - 4.22	
**Fatty Liver Index**				***< 0.001*** [(b)]
N-Miss	0	2	2	
Mean (SD)	67.32 (32.21)	17.42 (15.14)	42.37 (35.43)	
Median (Q1, Q3)	80.59 (48.81, 90.32)	13.64 (3.55, 30.53)	30.53 (10.39, 78.71)	
Range	3.35 - 98.61	1.32 - 42.42	1.32 - 98.61	

[(a)] Fisher exact test [(b)] Wilcoxon Rank Sum Test. In bold and italics, *p*-values < 0.05. Acronyms stand for: ALT, alanine aminotransferase; AST, aspartate aminotransferase; BMI, Body Mass Index; GGT, gamma-glutamyl transferase; HOMA, homeostasis model assessment; hsCRP, high-sensitivity C-reactive protein; IL-6, interleukin-6; TNF- α, tumor necrosis factor-α.

Table S3. Descriptive statistics on mitochondrial parameters at the baseline (T0).

Variables	MASLD (N=15)	Non_MASLD (N=17)	Total (N=32)	p value
**mtDNAcn**				0.133 (a)
N-Miss	0	1	1	
Mean (SD)	-0.28 (1.01)	0.26 (0.95)	-0.00 (1.00)	
Median (Q1, Q3)	-0.55 (-1.20, 0.60)	0.45 (-0.62, 1.09)	0.05 (-0.91, 1.01)	
Range	-1.49 - 1.20	-1.23 - 1.44	-1.49 - 1.44	
**Basal respiration**				***0.025*** (a)
Mean (SD)	-0.43 (0.69)	0.38 (1.09)	0.00 (1.00)	
Median (Q1, Q3)	-0.62 (-0.83, 0.03)	0.36 (-0.46, 1.16)	-0.20 (-0.75, 0.59)	
Range	-1.65 - 1.04	-1.47 - 2.16	-1.65 - 2.16	
**ATP production**				***0.030*** (a)
Mean (SD)	-0.42 (0.70)	0.37 (1.09)	-0.00 (1.00)	
Median (Q1, Q3)	-0.60 (-0.88, 0.02)	0.62 (-0.47, 0.96)	-0.11 (-0.66, 0.71)	
Range	-1.68 - 1.00	-1.56 - 2.12	-1.68 - 2.12	
**Maximal respiration**				***0.043*** (a)
Mean (SD)	-0.41 (0.77)	0.36 (1.06)	0.00 (1.00)	
Median (Q1, Q3)	-0.29 (-1.08, 0.24)	0.19 (-0.44, 1.08)	-0.02 (-0.84, 0.57)	
Range	-1.68 - 0.66	-1.06 - 2.34	-1.68 - 2.34	
**Spare respiratory capacity**				***0.047*** (a)
Mean (SD)	-0.38 (0.79)	0.33 (1.07)	0.00 (1.00)	
Median (Q1, Q3)	-0.22 (-1.10, 0.19)	0.15 (-0.72, 1.31)	0.01 (-0.75, 0.56)	
Range	-1.58 - 0.82	-1.02 - 2.39	-1.58 - 2.39	
**Proton leak**				0.206 (a)
Mean (SD)	-0.30 (0.61)	0.27 (1.20)	-0.00 (1.00)	
Median (Q1, Q3)	-0.42 (-0.84, 0.09)	-0.01 (-0.67, 0.45)	-0.24 (-0.72, 0.41)	
Range	-1.03 - 0.90	-1.03 - 2.94	-1.03 - 2.94	
**Non-mitochondrial respiration**				0.835 (a)
Mean (SD)	-0.11 (0.89)	0.09 (1.11)	0.00 (1.00)	
Median (Q1, Q3)	0.07 (-0.96, 0.52)	0.31 (-0.59, 0.39)	0.30 (-0.76, 0.52)	
Range	-1.38 - 1.29	-1.81 - 2.08	-1.81 - 2.08	

(a) Wilcoxon Rank Sum Test *p*-values. In bold and italics, *p*-values < 0.05. Acronyms stand for: mtDNAcn, mitochondrial DNA copy number.

Table S4. Descriptive statistics on mitochondrial parameters at follow-up (T12)

Variables	MASLD (N=15)	Non-MASLD (N=17)	Total (N=32)	p value
**mtDNAcn**				0.906 [(a)]
N-Miss	0	1	1	
Mean (SD)	-0.03 (0.69)	0.03 (1.25)	0.00 (1.00)	
Median (Q1, Q3)	0.04 (-0.48, 0.13)	-0.02 (-0.65, 0.41)	-0.01 (-0.61, 0.31)	
Range	-1.20 - 1.31	-2.02 - 3.50	-2.02 - 3.50	
**Basal respiration**				0.865 [(a)]
Mean (SD)	-0.15 (0.63)	0.13 (1.24)	0.00 (1.00)	
Median (Q1, Q3)	-0.17 (-0.38, 0.23)	-0.21 (-0.66, 0.97)	-0.19 (-0.60, 0.49)	
Range	-1.36 - 0.85	-1.62 - 2.53	-1.62 - 2.53	
**ATP production**				0.835 [(a)]
Mean (SD)	-0.15 (0.70)	0.13 (1.21)	-0.00 (1.00)	
Median (Q1, Q3)	-0.18 (-0.36, 0.34)	-0.41 (-0.74, 0.96)	-0.19 (-0.59, 0.65)	
Range	-1.52 - 0.85	-1.65 - 2.37	-1.65 - 2.37	
**Maximal respiration**				0.610 [(a)]
Mean (SD)	-0.02 (0.71)	0.02 (1.22)	-0.00 (1.00)	
Median (Q1, Q3)	-0.14 (-0.53, 0.57)	-0.33 (-0.77, 0.55)	-0.21 (-0.73, 0.56)	
Range	-1.14 - 1.17	-1.12 - 3.20	-1.14 - 3.20	
**Spare respiratory capacity**				0.439 [(a)]
Mean (SD)	0.02 (0.73)	-0.02 (1.21)	0.00 (1.00)	
Median (Q1, Q3)	-0.16 (-0.45, 0.65)	-0.53 (-0.88, 0.68)	-0.34 (-0.69, 0.69)	
Range	-1.27 - 1.18	-1.23 - 3.17	-1.27 - 3.17	
**Proton leak**				0.417 [(a)]
Mean (SD)	-0.14 (0.32)	0.13 (1.35)	0.00 (1.00)	
Median (Q1, Q3)	-0.20 (-0.35, -0.12)	-0.36 (-0.74, 0.51)	-0.25 (-0.47, 0.02)	
Range	-0.59 - 0.59	-0.94 - 4.06	-0.94 - 4.06	
**Non-mitochondrial respiration**				0.850 [(a)]
Mean (SD)	-0.07 (1.00)	0.06 (1.03)	-0.00 (1.00)	
Median (Q1, Q3)	-0.12 (-0.71, 0.59)	-0.15 (-0.70, 0.72)	-0.12 (-0.71, 0.65)	
Range	-1.64 - 2.00	-1.25 - 2.29	-1.64 - 2.29	

[(a)] *p*-values of the Wilcoxon Rank Sum Test. The acronym stands for: mtDNAcn, mitochondrial DNA copy number.

Table S5. Coefficients of a Linear Mixed-effect Model on mitochondrial parameters evaluated over time

**MASLD**			
**Variables**	**Coefficients**	**Standard Error**	**p-value**
mtDNAcn	0.25	0.32	0.439
Basal respiration	0.28	0.20	0.185
ATP production	0.27	0.21	0.227
Maximal respiration	0.39	0.27	0.162
Spare respiratory capacity	0.40	0.28	0.165
Proton leak	0.16	0.18	0.376
Non-mitochondrial respiration	0.03	0.32	0.908
**Non-MASLD**			
**Variables**	**Coefficients**	**Standard Error**	**p-value**
mtDNAcn	-0.23	0.35	0.512
Basal respiration	-0.24	0.39	0.542
ATP production	-0.24	0.40	0.560
Maximal respiration	0.39	0.27	0.162
Spare respiratory capacity	-0.35	0.39	0.379
Proton leak	-0.14	0.43	0.747
Non-mitochondrial respiration	-0.03	0.37	0.928

A Linear Mixed-effects Model (LMM) was estimated using the Restricted Maximum Likelihood (REML) method; p-values are computed with the *t*-tests based on Satterthwaite's approximation. The table contains the coefficients representing the mean difference of each mitochondrial parameter computed over time. The analysis is repeated for MASLD patients and non-MASLD subjects. Positive values indicate that, on average, the parameters tend to increase over time, while negative values suggest the opposite. Since all p-values exceed 0.05, these changes are not statistically significant.

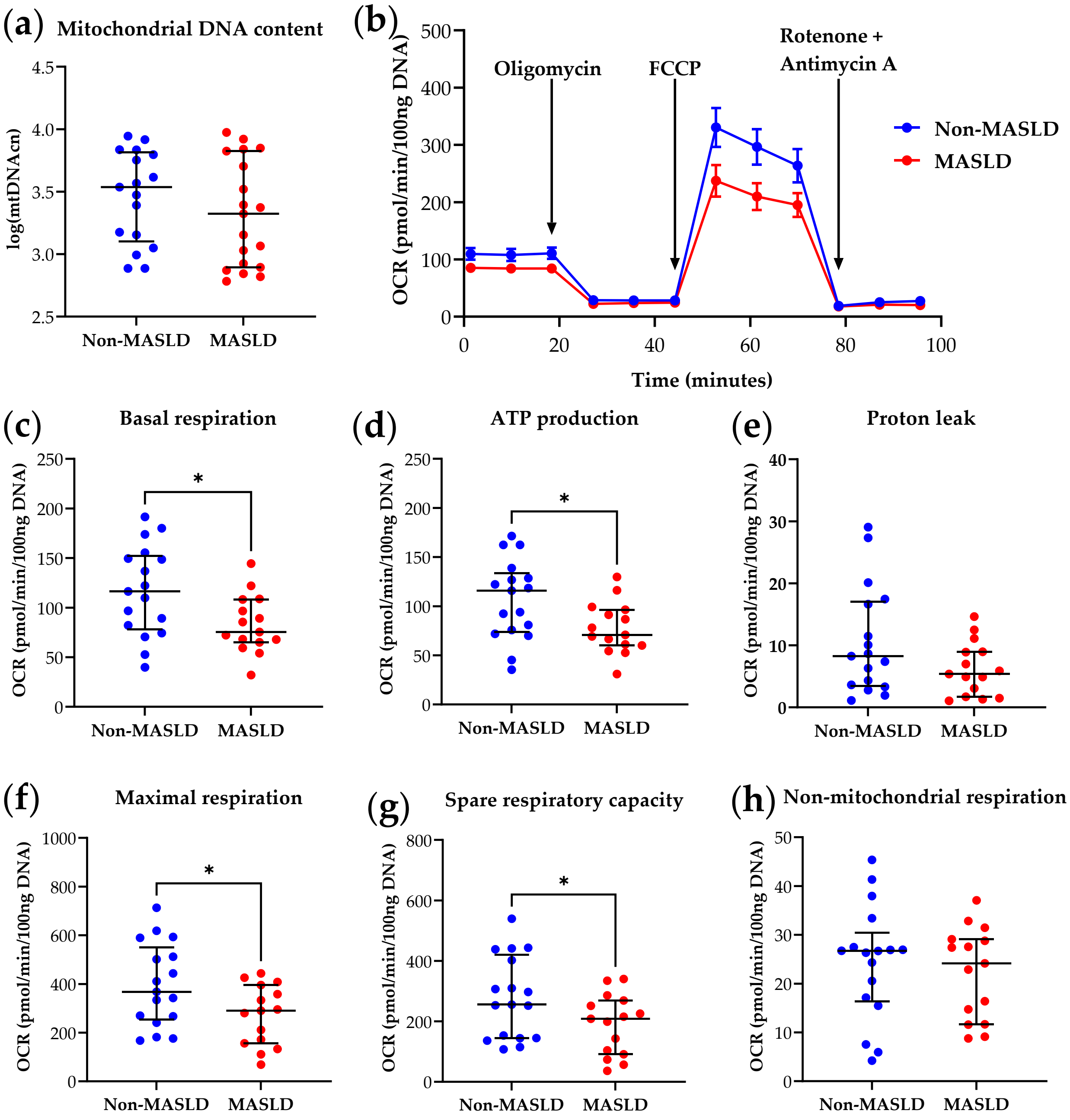

Figure S1. Mitochondrial content and function in PBMCs from MASLD patients and non-MASLD controls at the baseline (T0). (**a**) quantification of mitochondrial DNA copy number (mtDNAcn) with values log-transformed. (**b**) Real-time course of oxygen consumption rate (OCR) measured in live PBMCs during the Mito Stress Test using the Seahorse XFe24 analyzer. Three measurements were performed at different times for each condition. OCR values were normalized to DNA content. (**c**-**h**) Scatter dot plots of OCR values from individual subjects, normalized to DNA content, for each respiratory parameter. Data represent median ± interquartile range (**a**; **c**-**h**) or mean ± SEM (**b**). Wilcoxon Rank Sum test was applied (* p-values <0.05). Graphs were performed using Prism 9.0.0 (GraphPad).
